# Degradation mechanisms of silver nanowire electrodes under ultraviolet irradiation and heat treatment

**DOI:** 10.1038/s41598-017-01843-9

**Published:** 2017-05-10

**Authors:** Dong Chul Choo, Tae Whan Kim

**Affiliations:** Department of Electronics and Computer Engineering, Hanyang University, 222 Wangsimni-ro, Seongdong-gu, Seoul, 04763 Korea

## Abstract

We report the degradation mechanisms of the silver nanowire (Ag NW) electrodes that play a significantly important role in the stability of wearable and flexible devices. The degradation mechanisms behind the increase in the sheet resistances of Ag NW electrodes were clarified by investigating the variations in the structure and the chemical composition of the Ag NW electrodes caused by ultraviolet irradiation and thermal treatment. While the shapes of the Ag NWs were affected by melting during the thermal degradation process, the chemical composition of the polyvinylpyrrolidone protective layer on the surfaces of the Ag NWs was not changed. Ultraviolet irradiation deformed the shapes of the Ag NWs because nitrogen or oxygen atoms were introduced to the silver atoms on the surfaces of the Ag NWs. A graphene-oxide flake was coated on the Ag NW electrodes by using a simple dipping method to prevent ultraviolet irradiation and ozone contact with the surfaces of the Ag NWs, and the increase in the sheet resistance in the graphene-oxide-treated Ag NWs was suppressed. These observations will be of assistance to researchers trying to find novel ways to improve the stability of the Ag NW electrodes in next-generation wearable devices.

## Introduction

Research on the formation of transparent electrodes and nanocomposite active layers for next-generation flexible optoelectronic applications by using graphene and graphene-related materials^1–4^, carbon nanotubes^5–7^, polymers^8–10^ and silver nanowires (Ag NWs) has been actively conducted^11–15^. Ag NW electrodes have been attracting a great deal of concerns due to their having high flexibility, transparency, electrical conductivity, and low cost among the various alternative flexible electrodes^11–15^. While almost all candidates for alternative transparent electrodes show excellent flexibility in comparison with the most widely used indium-tin-oxide (ITO) electrodes, their sheet resistances, transmittances, and production costs still do not match those of ITO films^16, 17^. Some mobile phones that use rigid plastic substrates have already been released, and electronic devices based on flexible substrates are expected to be commercialized in the near future^18^. However, research on flexible, transparent, conductive electrode candidates that can completely replace ITO electrodes seems to have encountered a confusing situation^19–21^. Among the flexible, transparent, conductive electrode candidates, Ag NW electrodes have already been applied to some commercial products and have exhibited great advantages in cost due to the rapid development of synthesis and production technologies^22–26^. Flexible Ag NW electrodes have been studied in a wide variety of applications as electrodes for flexible touch panels, organic light-emitting devices, organic photovoltaic devices, and nanogenerators and are considered to be the most likely candidates for flexible, transparent, conductive electrodes if no breakthroughs in other candidates occur. However, Ag NW electrodes are known to be very vulnerable to heat, oxygen, moisture, and light^27–32^, and they have the potential problem that due to these disadvantages, their physical properties may deteriorate very rapidly during the product manufacturing process or during the use of the product. Even though several studies have reported on deterioration phenomena in electronic and optoelectronic devices caused by moisture or heat, systematic investigations concerning the underlying origins of the degradations of Ag NWs and the degradation mechanisms are necessary to enhance the lifetimes and the stabilities of Ag NWs^27–32^.

In this research, the mechanisms underlying the degradation of Ag NWs due to ultraviolet irradiation and thermal treatment were investigated based on the electrical, the optical, the chemical, and the structural properties of the Ag NWs. The electrical and the optical properties of the Ag NW electrodes were investigated by measuring their sheet resistances and absorption spectra. The structural properties of the Ag NWs were investigated by using scanning electron microscopy (SEM) and transmission electron microscopy (TEM) measurements. The variations in the chemical composition on the surfaces of the Ag NWs due to ultraviolet irradiation and thermal treatment were analyzed using X-ray photoelectron spectroscopy (XPS) and energy dispersive X-ray spectroscopy (EDS) measurements.

## Methods

### Sample preparation

Soda-lime glass and silicon (Si) substrates were cleaned by using acetone and methanol for 10 min each in sequence and were then thoroughly rinsed in de-ionized water. Next, the chemically cleaned glass and Si substrates were dried by using N_2_ gas, and the surfaces of the substrates were treated with ultraviolet/ozone (UV/O_3_) treatment for 20 min. An Ag NW solution was spin-coated onto the cleaned substrates, resulting in the formation of uniform and transparent electrodes. Ag NWs were dispersed in isopropyl alcohol at a concentration of 0.5 wt% (purchased from Nanopyxis Co.). The typical length and diameter of the Ag NWs were 25 μm and 30 nm, respectively. The spin-coating speed and time were 6000 rpm and 30 sec, respectively, and the Ag NW solution was dropped by using a micropipette with a volume between 10 and 20 μl. After the Ag NW solution had been coated on the substrates, the substrates were dried for 1 hr in an atmospheric environment. The Ag NW electrodes were thermally treated for 10 min under an atmospheric environment after a certain temperature had been set on the hot plate because the sheet resistance of the Ag NW electrodes was stabilized after a 10-min thermal treatment. After a preliminary experiment, the thermal treatment time was set as the time required for sheet-resistance stabilization in the Ag NW electrodes. The thermal treatment temperature for the Ag NW electrodes was varied from 85 to 210 °C. The UV/O_3_ treatment was performed by using a UV/ozone cleaner to expose the Ag NW electrodes to UV radiation at wavelengths of 185 and 254 nm.

### Electrical, optical, chemical, and structural measurements

The sheet resistances were measured using a sheet resistance meter (FPP-40K, DASOL ENG) and were obtained by measuring nine points on the substrate and determining the mean value and the standard deviation. The absorption spectra were measured by using an ultraviolet-visible spectrometer (Lambda 650S, Perkin Elmer). The surface chemical compositions were measured by using XPS (XPS-Theta Probe, Thermo Fisher Scientific Co.). The structure and the chemical composition were investigated by using SEM (NOVA NANOSEM 450, FEI), TEM, and EDS (JEM-2100F, JEOL).

## Results

**Figure 1:**
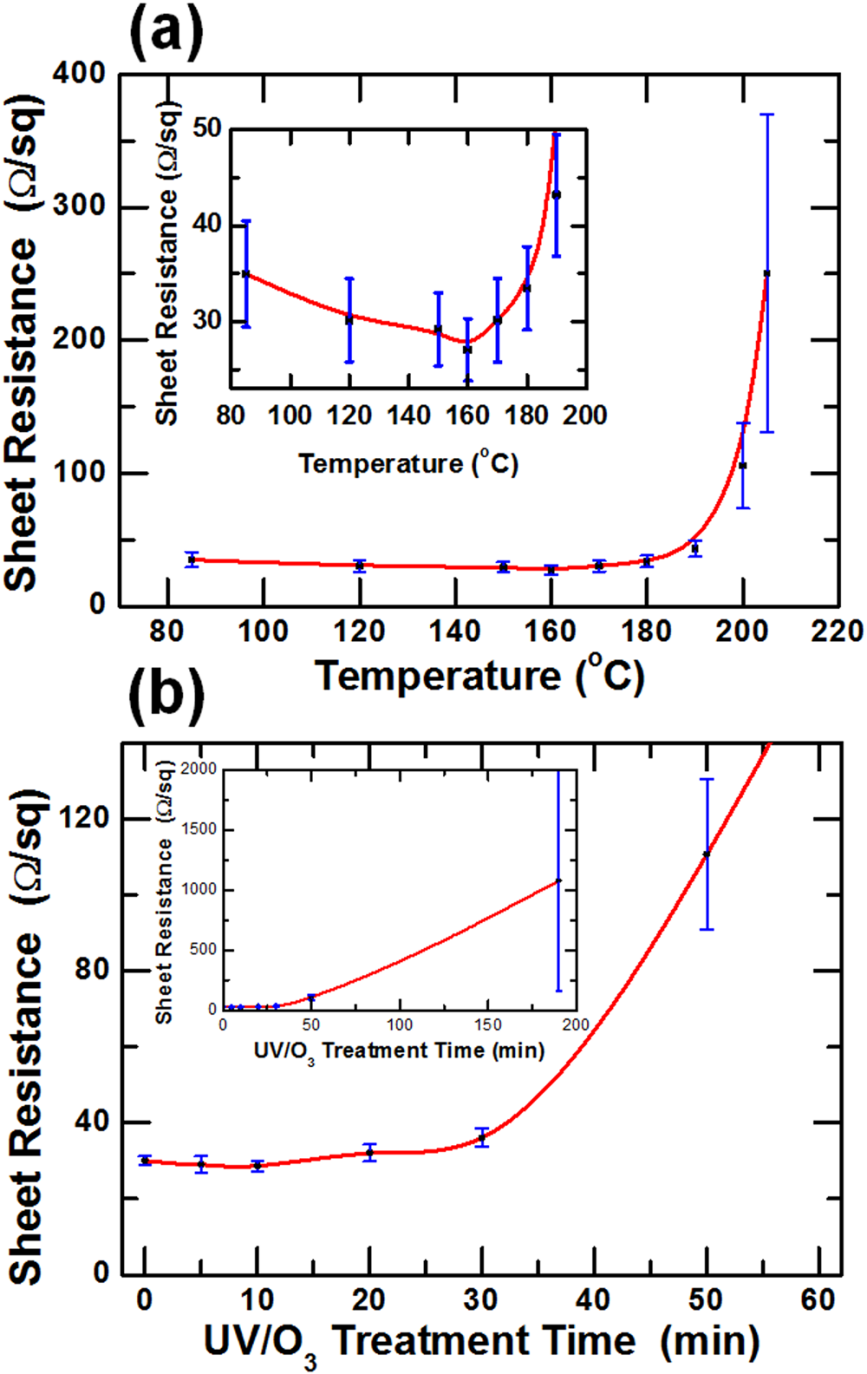
Sheet resistance of the Ag NW electrodes as functions of the (**a**) thermal treatment temperature and the (**b**) UV/O_3_ treatment time.

Figures 1(a) and (b) show the sheet resistances as functions of the thermal treatment temperature and the UV/O_3_ treatment time. The sheet resistance of the Ag NW electrodes decreased slightly when the thermal treatment temperature was below 160 °C, as shown in Fig. 1(a). However, the sheet resistance gradually increased with increasing temperature until about 200 °C, at which point a dramatic increase in the sheet resistance with a further increase in temperature was observed. Finally, the sheet resistance of the Ag NW electrodes at temperatures above 205 °C could not be measured. Even though the melting point of silver is approximately 962 °C, the melting point of Ag NWs is dramatically lower due to the high surface stress originating from the nanostructures^33, 34^. The Ag NWs melted at relatively low temperature of 85 °C, resulting in a decrease in the contact resistances between Ag NWs. However, the melting of the surface the Ag NWs caused their percolation network to be broken when the thermal treatment temperature was increased to temperatures above 160 °C, resulting in an increase in the sheet resistance. Because the percolation network of the Ag NW electrodes was completely destroyed at temperatures above 205 °C, the sheet resistances of the thermally treated Ag NW electrodes could not be measured. Figure 1(b) shows the sheet resistance as a function of the UV/O_3_ treatment time at room temperature. The sheet resistance of the Ag NW electrodes slightly fluctuated with the UV/O_3_ treatment time for treatment times up to 30 min. However, the sheet resistance of the Ag NW electrodes significantly increased with increasing UV/O_3_ treatment time for treatment times longer than 30 min, and the standard deviation also increased. After a UV/O_3_ treatment of 180 min, the average sheet resistance and its standard deviation were approximately 1079 and 921 Ω/sq, respectively. Furthermore, six of the nine measurements were not available during the measurements for the sheet resistances of the Ag NW electrodes because the percolation network of the Ag NWs was broken by the UV/O_3_ treatment. The degradation mechanisms generated by the UV and the thermal treatments needed to be clarified separately because the temperature of the Ag NW electrodes was increased up to 36 °C in the UV/O_3_ cleaner, a temperature at which thermal deterioration was barely observed.

**Figure 2:**
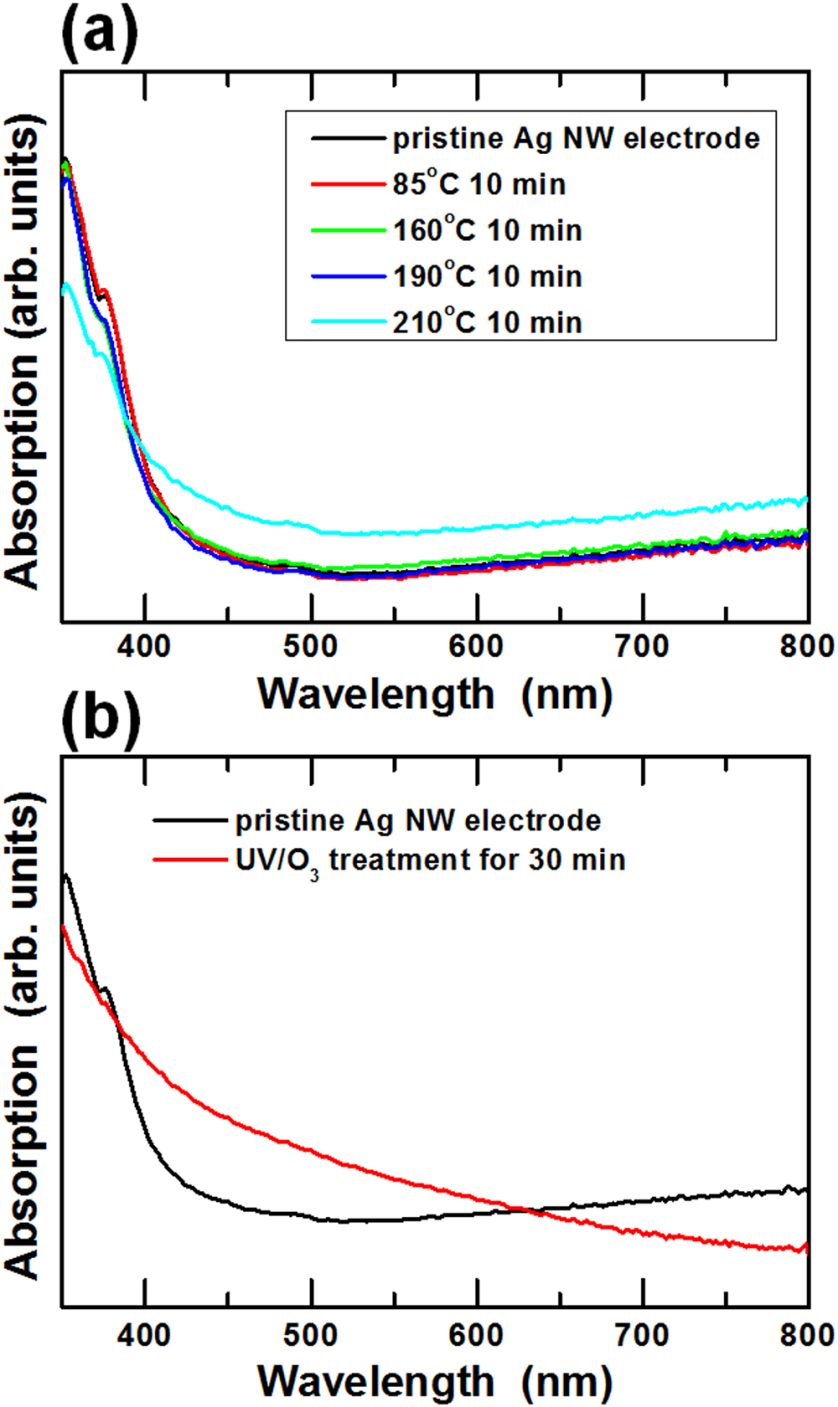
Absorption spectra of the Ag NW electrodes for (**a**) a 10-min thermal treatment at various temperatures and for (**b**) a 30-min UV/O_3_ treatment.

**Figure 3:**
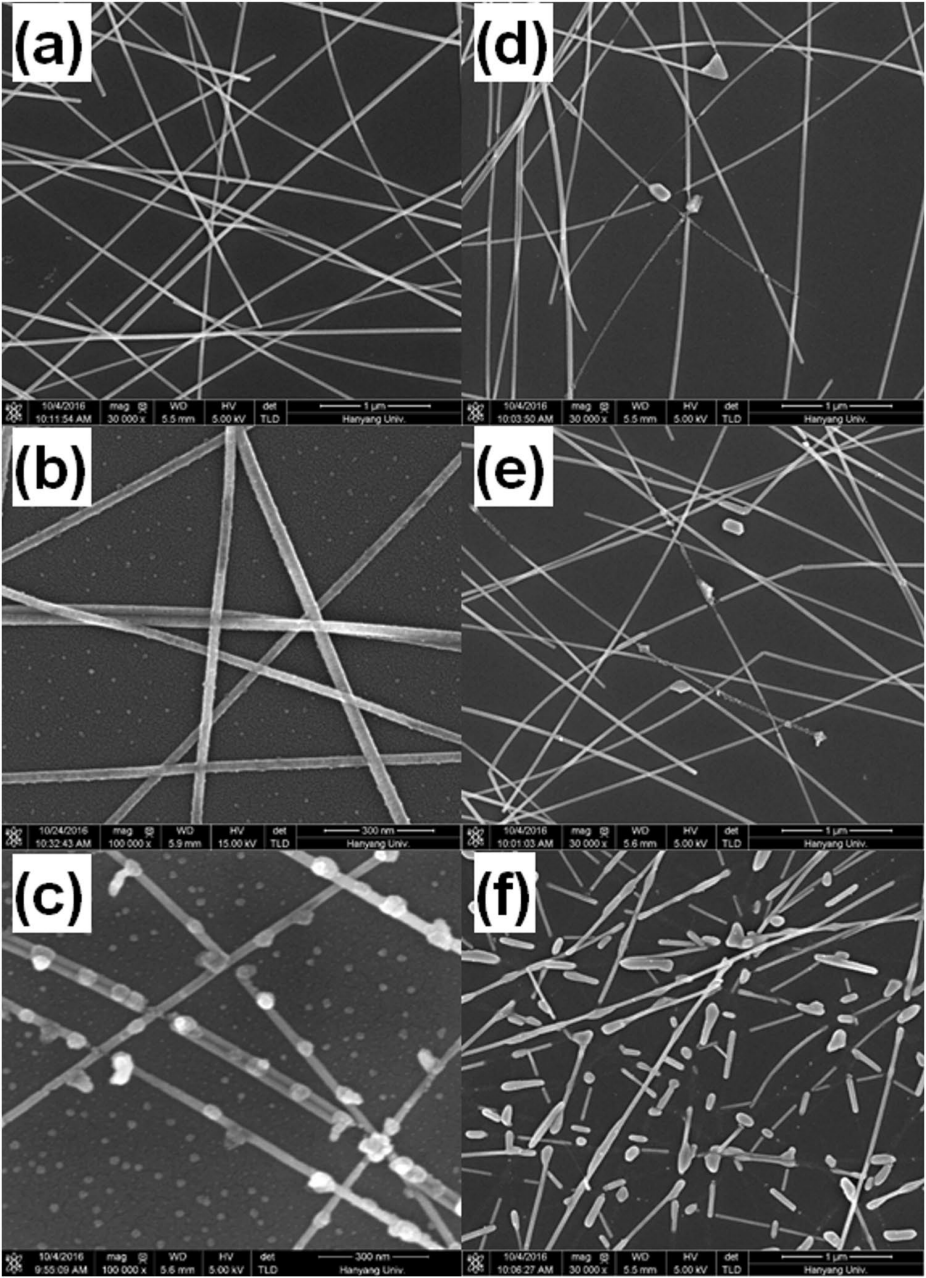
Scanning electron microscopy images of the (**a**) pristine Ag NWs, the (**b**) Ag NWs UV/O_3_-treated for 30 min, the (**c**) Ag NWs UV/O_3_-treated for 60 min, and the Ag NWs thermally treated at (**d**) 160, (**e**) 190, and (**f**) 210 °C for 10 min on Si substrates.

Figures 2(a) and (b) show the absorption spectra for the thermally-treated and the UV/O_3_-treated Ag NW electrodes. Two extinction peaks, one around 350 nm and the other around 380 nm were observed in the pristine Ag NW electrode, and these peaks were associated with the quadruple resonance excitation that is typically related to the longitudinal surface plasmon resonance and the transverse surface plasmon resonance (TSPR) of the Ag NWs^35, 36^. The TSPR peak disappeared at 160 °C and appeared again at 210 °C, as shown in Fig. 2(a). The variation in the TSPR peak was attributed to a change in the shape of the Ag NWs. Figure 2(b) shows the shrinkage of the TSPR peak due to the UV/O_3_ treatment. The variation in the TSPR peak for the Ag NWs could be described based on the structural measurements for the Ag NWs, which are shown in Fig. 3.

Figure 3 shows SEM images for the pristine Ag NWs, the UV/O_3_-treated Ag NWs, and the thermally-treated Ag NWs. While the Ag NW electrodes were coated on a glass substrate to form a transparent electrode, the Si substrates were used for SEM measurements in order to avoid observation difficulties due to charge accumulation on the substrate. However, because the electrical characteristics of the Ag NWs formed on the Si substrate with a 100-nm-thick SiO_2_ layer were not affected by the Si substrate, the adhesive force, the dispersion, and the degradation progress of the Ag NWs were independent on the type of substrate. While the pristine Ag NWs shown in Fig. 3(a) had smooth surfaces, small protrusions were generated on the surfaces of the Ag NWs with increasing UV/O_3_ treatment time, as shown in Fig. 3(b). As the time of exposure to UV light increased, the Ag NWs broke at the protrusions, as shown in Fig. 3(c). Figure 3(d)–(f) show that the structural properties of the Ag NWs changed with increasing thermal treatment temperature. When the thermal treatment temperature was 160 °C, a few regions around the contact points of the Ag NWs melted due to the applied thermal energy. Many parts of the Ag NWs melted with increasing thermal treatment temperature, and the structure of the Ag NWs was transformed into the sphere-type structure. The variation in the TSPR peak for the Ag NWs, as shown in Fig. 2(a), could be explained by using the variation in the shape of the Ag NWs due to the increase in the thermal treatment temperature. Because the TSPR of the Ag NWs originated from the plasmon resonance along the thickness direction, an increase in the melted area of the Ag NWs suppressed the TSPR, resulting in a decrease in or disappearance of the TSPR peak in the absorption spectra, as shown in Fig. 2(a). However, when the thermal treatment temperature was raised to 210 °C, spherically-shaped particles were generated, and the TSPR absorption peak reappeared due to the plasmon resonance of those particles, as shown in Fig. 3(f). Even though the intensity of the quadruple resonance excitation peak decreases with increasing thermal treatment temperature, the peak is still present. The SEM image in Fig. 3(f) shows that even though many Ag NWs were separated by the thermal treatment, resulting in the formation of nanoparticle-like particles, short rod-shaped Ag NWs were still present. The quadrupole resonance excitation, which is the longitudinal plasmon resonance mode of the Ag NWs, appears to be derived from the rod-shaped Ag NW.

**Figure 4:**
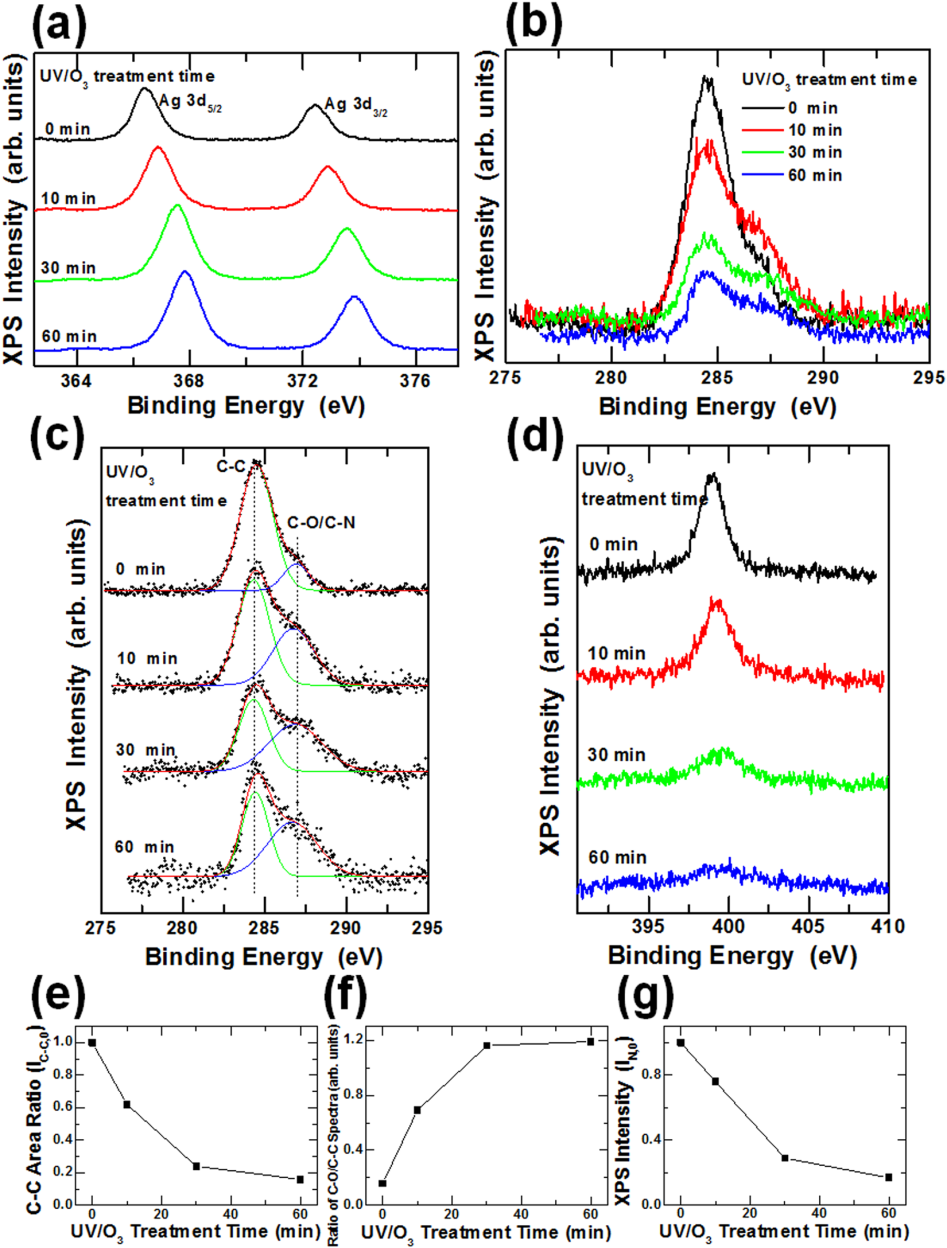
X-ray photoelectron spectroscopy spectra of the Ag NWs heat-treated for 0, 10, 30, and 60 min: (**a**) Ag 3d, (**b**) C 1s, (**c**) C 1s. (**d**) N 1s spectra of the Ag NWs heat-treated for 0, 10, 30, and 60 min. (**e**) Normalized C-C spectral area as a function of the UV/O_3_ treatment time, and (**f**) C-O/C-N spectral area ratio based on the C-C spectral area as a function of the UV/O_3_ treatment time. (**g**) N 1s peak intensity as a function of the UV/O_3_ treatment time.

**Figure 5:**
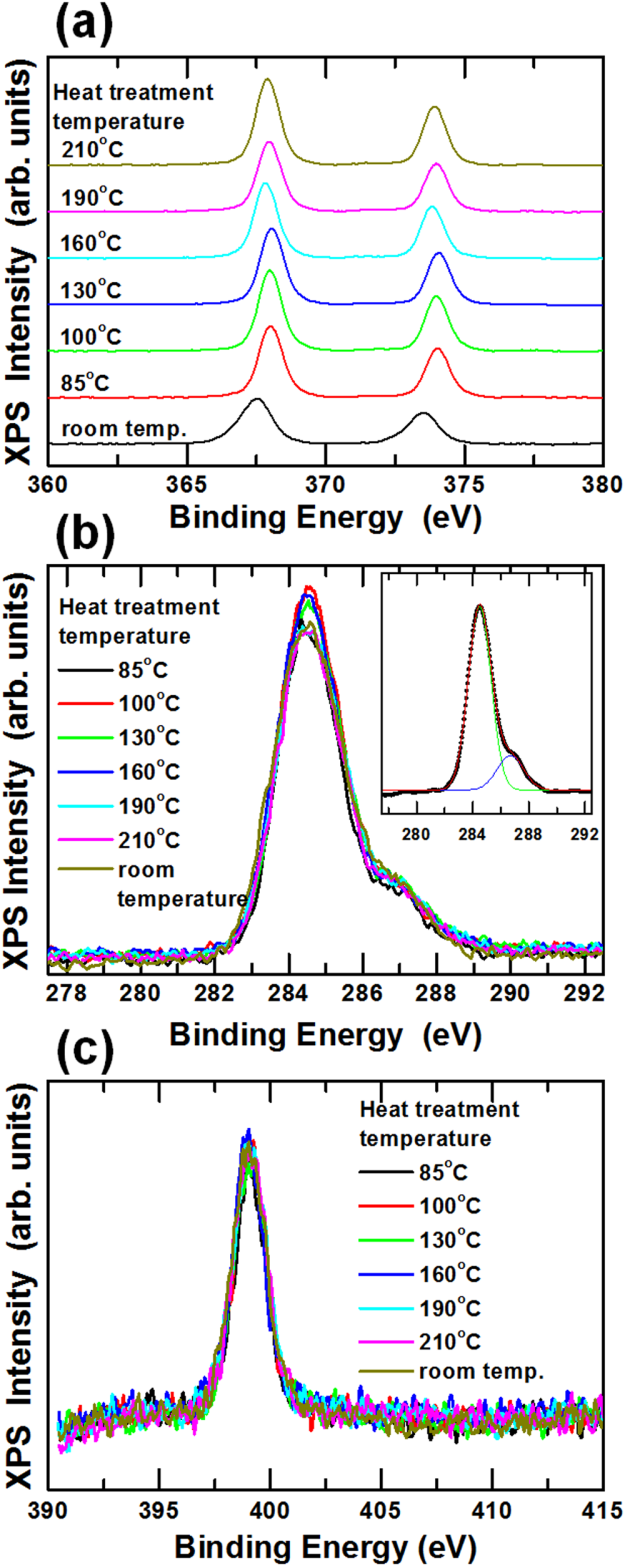
X-ray photoelectron spectroscopy spectra of the Ag NWs thermally-treated at room temperature, 85, 100, 130, 160, 190, and 210 °C for 10 min: (**a**) Ag 3d, (**b**) C 1s, and (**c**) N 1s spectra.

Figure 4 shows the XPS spectra for the pristine Ag NWs and the UV/O_3_-treated Ag NWs. Ag NWs without a PVP protective layer might be a good reference for investigating the surface composition variation. However, synthesizing Ag NWs without a PVP protective layer and forming Ag NW electrodes are difficult because PVP is an essential material for the anisotropic formation of Ag NWs and for preventing aggregation of those Ag NWs. The Ag 3d peaks of the Ag NWs shifted to higher binding energy with increasing UV/O_3_ treatment time, as shown in Fig. 4(a). Because the binding energy of the Ag atoms in a metal phase was larger than that of Ag atoms in an oxide phase, the peak shift resulting from the UV/O_3_ treatment implies a reduction in the amount of Ag oxide on the surfaces of the Ag NWs^37, 38^. The intensity of the C 1s peaks for the Ag NWs decreased with increasing UV irradiation time, as shown in Fig. 4(b). The C 1s spectra were deconvoluted into two components, C-C and C-O/C-N bonds, to investigate the variations in the C 1s spectra. Figure 4(c) shows the deconvolution spectra of the C 1s spectra for the pristine and the UV/O_3_-treated Ag NWs. The areas corresponding to the C-C bonds decreased with increasing UV/O_3_ treatment time, as shown in Fig. 4(e), and the C-C spectral area of the pristine Ag NWs decreased by 70% within 30 min. This result showed that about 70% of the C was removed within 30 min due to the UV irradiation. The ratios of the C-C to the C-O/C-N spectral areas for the Ag NWs UV/O_3_-treated for 0, 10, 30, and 60 min were 1:0.16, 1:0.69, 1:1.16, and 1:1.19, respectively. The ratios of the C-O/C-N spectral area relative to the C-C spectral area in Fig. 4(c) were plotted in Fig. 4(f). The ratios of the C-O to the C-N spectral area were seen to increase dramatically with increasing UV/O_3_ treatment times up to 30 min. Because the C-O and the C-N spectra could not be divided easily due to the overlap of their peak positions, they were treated as a full peak. However, the N peak’s intensity decreased rapidly for treatment times up to 30 min, and only 17% of the initial peak intensity was detected after a treatment time of 60 min, as shown in Fig. 4(g). The initial decrease in the C-O/C-N spectra dominantly resulted from a decrease in the nitrogen content, and the C-O/C-N spectra remaining after 60 min was mainly attributed to the oxygen content. The variations with UV/O_3_-treatment time in the intensities for the N spectra in Fig. 4(d) are shown in Fig. 4(g). These XPS results demonstrate that UV treatment changed the phase of the Ag atoms in the Ag NWs from the oxide phase to the metal phase, resulting in a decrease in the numbers of carbon and nitrogen atoms. Figure 5 shows the XPS spectra for the thermally-treated Ag NWs. The Ag 3d peaks shifted to a higher binding energy due to the thermal treatment. This behavior originated from the phase transition of the Ag atoms on the surfaces of the Ag NWs from the oxide phase to the metal phase. However, the positions of the C 1s and the N 1s peaks hardly changed with varying thermal treatment temperature. These results demonstrate that the chemical composition of the Ag NWs did not change during the thermal treatment; only their morphology had been deformed.

**Figure 6:**
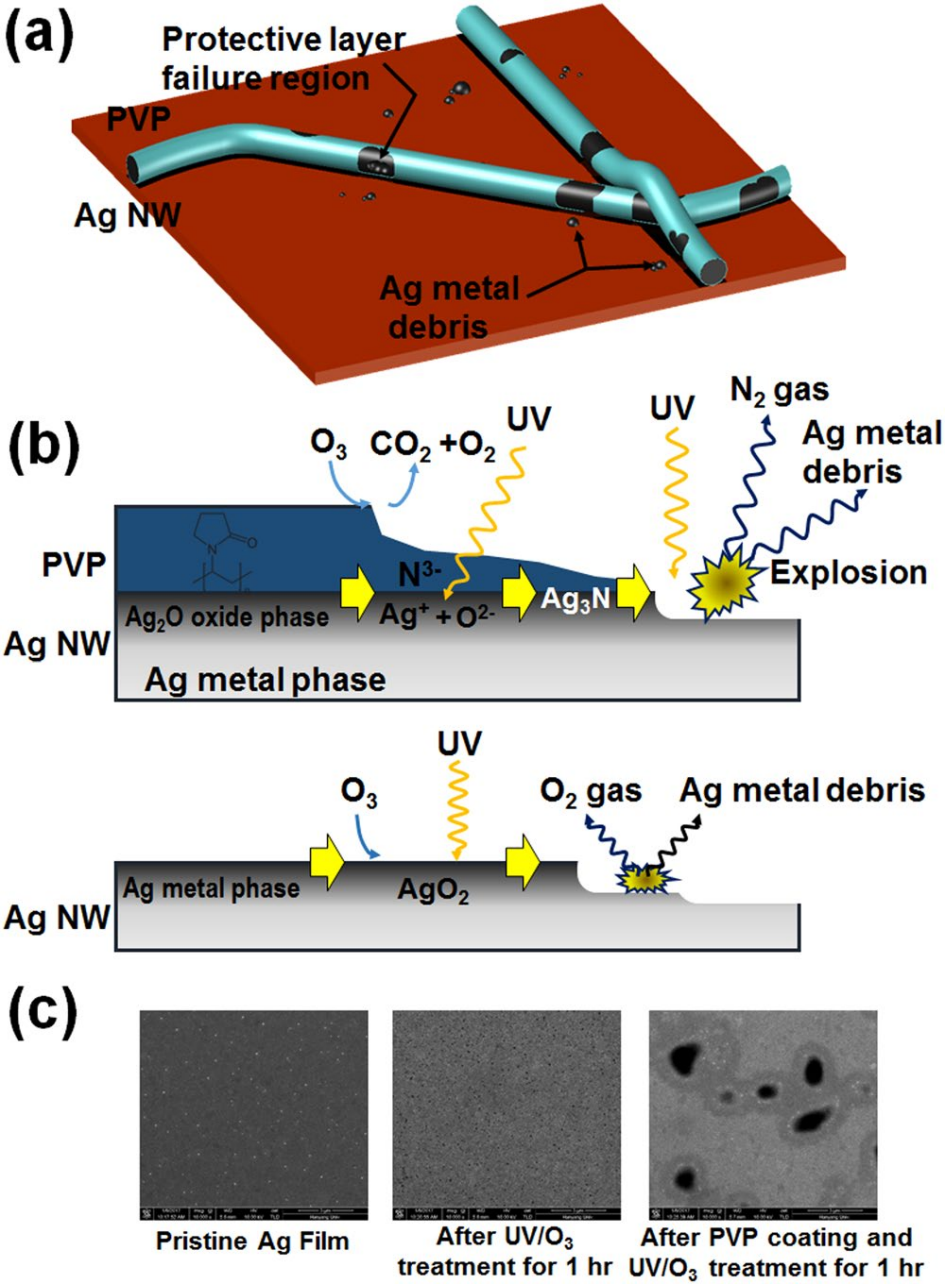
Schematic diagrams for the (**a**) cross-sectional images of the Ag NWs and for the (**b**) chemical reactions on the surfaces of the Ag NWs. (**c**) Scanning electron microscopy images for the pristine silver film with a 100-nm thickness, for the silver film after UV/O_3_ treatment for 1 hr, and for the silver films after polyvinylpyrrolidone coating and UV/O_3_ treatment for 1 hr.

Figure 6 shows schematic diagrams for the degradation process of Ag NWs irradiated by UV light. A polyvinylpyrrolidone (PVP) protecting layer with a thickness of about 1 nm surrounded the Ag NWs, and some Ag atoms on the surfaces of Ag NWs were in the oxide state, which was confirmed by using the XPS spectra. The PVP plays an important role in controlling the anisotropic growth of the Ag NWs during their synthesis and in preventing severe aggregation of the synthesized Ag NWs^39^. However, the UV rays irradiating the surfaces of the Ag NWs decomposed the silver oxide on those surfaces to produce silver and oxygen ions. The same decomposition occurred even in the PVP layer, resulting in the formation of nitrogen ions. Because the binding energy of C-N bonds was smaller than those of C=O and C-C bonds, the probability of breaking the C-N bonds was relatively high. The Ag and the nitrogen ions combined to form silver nitride at the interface of the Ag NW and the PVP protective layer. Because silver nitride is an explosive chemical compound and decomposes explosively to metallic silver and nitrogen gas at temperatures between 160 and 320 °C^40, 41^, the silver nitride on the surfaces of the Ag NWs caused small explosion, thus deforming the shapes of the Ag NWs. The energy causing the small explosion was suspected to have come from the UV light. The shapes of the Ag NWs were transformed due to the small explosions, as shown in Fig. 3(b) and (c). However, such a transformation of the shapes of the Ag NWs continued even after the disappearance of the nitrogen atoms. A binding reaction similar to the reaction between the nitrogen and the silver atoms proceeded with the oxygen and the silver atoms, and the resulting silver oxide was decomposed because of the UV light^42, 43^. Even though the explosive force at the time of the decomposition of silver oxide was smaller than that of silver nitride, the surfaces of the Ag NWs were continuously deformed to form silver fragments around the NWs. The deformation caused by the UV/O_3_ treatment of the silver surface was also observed in the silver film, which was confirmed through UV/O_3_ treatment experiments on the silver film and the PVP-coated silver film^44^, the results of which are shown in Fig. 6(c). The SEM images showed that small holes were formed in the surface of the silver film due to the UV treatment for 1 hr. However, very large pores were created on the surface of the Ag film coated with a PVP layer containing N atoms, and the average size of pores was almost a few μm.

**Figure 7:**
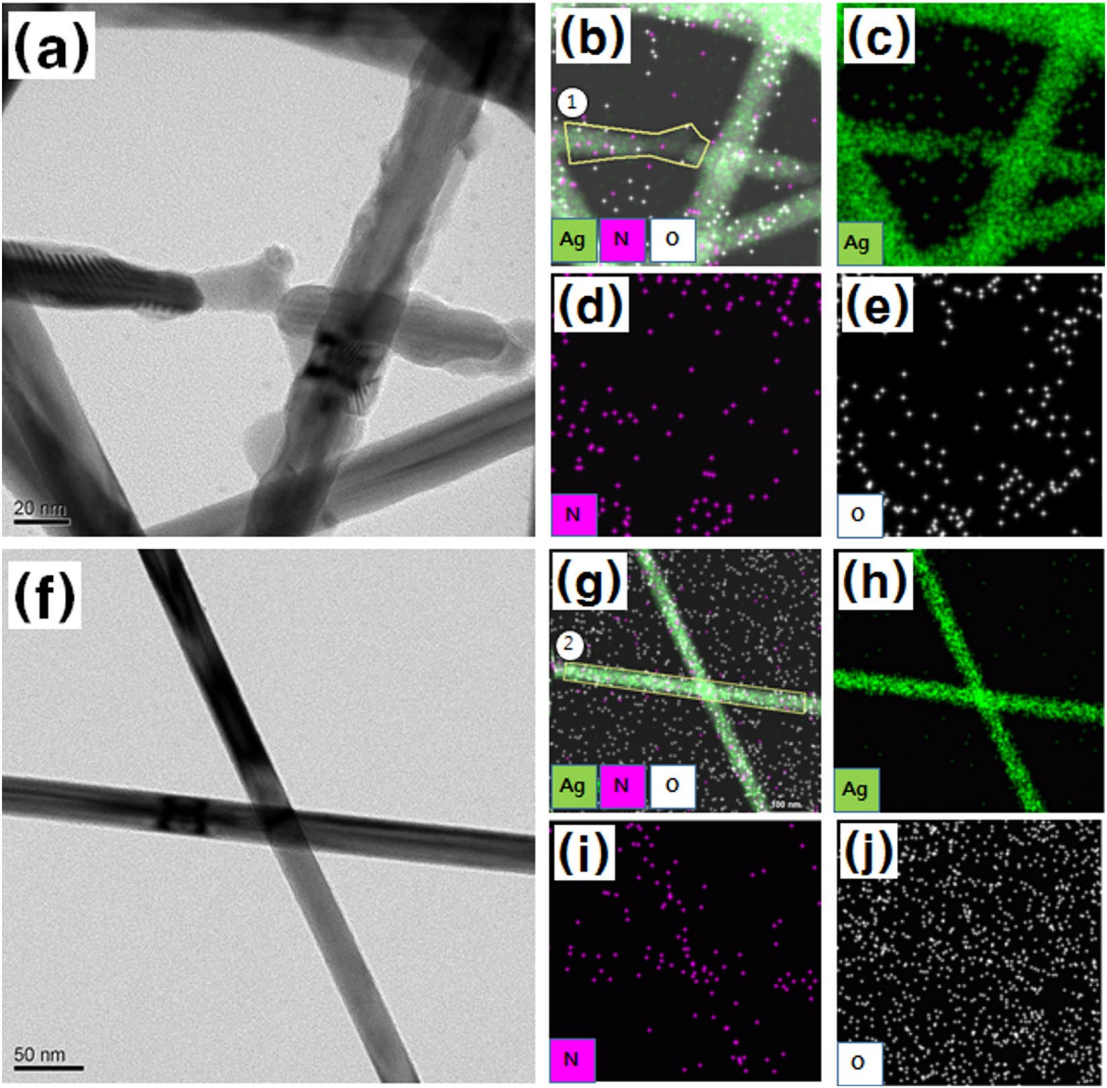
Transmission electron microscopy (TEM) image for the (**a**) UV/O_3_-treated Ag NWs, the (**b**) combined energy dispersive X-ray spectroscopy (EDS) map, and the EDS maps for (**c**) Ag, (**d**) N, and (**e**) O atoms for the Ag NWs UV/O_3_-treated for 30 min, and TEM image for the (**f**) pristine Ag NWs, the (**g**) combined EDS map, and the EDS maps for (**h**) Ag, (**i**) N, and (**j**) O for the pristine Ag NWs.

The TEM images of the Ag NWs treated by using UV light for 30 min and the pristine Ag NWs showed a variation in the components on the surfaces of the Ag NWs, as shown in Fig. 7. Region 1 in Fig. 7(b) and region 2 in Fig. 7(g) are regions containing degraded and non-degraded Ag NWs, respectively. While the nitrogen and the oxygen components observed in region 1 were very small, those observed in region 2 were not. The atomic percents of the components in regions 1 and 2 are summarized in Table 1. The atomic composition of the Ag NWs deformed during the UV/O_3_ treatment for 30 min was 100% silver while that of the pristine Ag NWs was nitrogen and oxygen atoms, together with silver atoms. The Ag ions generated by the UV irradiation reacted with the nitrogen contained in the PVP to generate nitrogen gas, thus reducing the amount of nitrogen in the PVP, and reacted with oxygen in a nitrogen-deficient environment to form silver oxide, which was decomposed by the UV irradiation. The shapes of the Ag NWs were deformed by the formation and the decomposition of the silver nitride and the silver oxide, and small pieces of debris remained around the Ag NWs, as shown in Fig. 3(b) and (c).

**Table 1:** Atomic ratios measured by using energy dispersive X-ray spectroscopy on regions 1 and 2.

Region	N (%)	O (%)	Ag (%)
1	0.00	0.00	100.00
2	0.88	14.68	84.43

**Figure 8:**
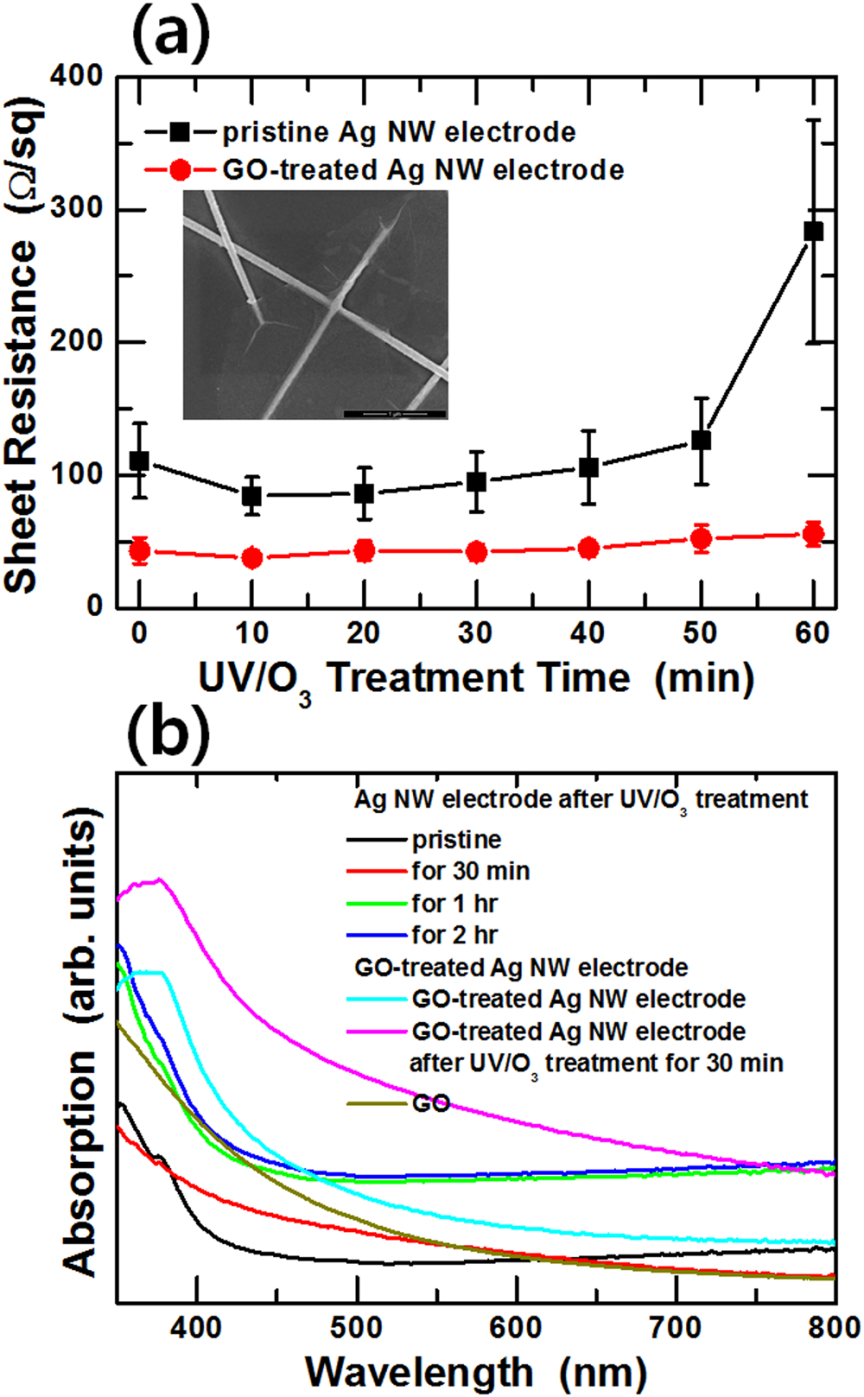
(**a**) Sheet resistance variations of the Ag NW electrodes with and without a GO treatment for various UV/O_3_ treatment times. (**b**) Absorption spectra of the pristine Ag NWs, the Ag NWs after UV/O_3_ treatment for various times, the graphene oxide (GO)-treated Ag NWs, and the GO-treated Ag NWs after UV/O_3_ treatment for 30 min.

Figure 8 shows the (a) sheet resistance as a function of the UV/O_3_ treatment time and the (b) absorption spectra of the pristine Ag NWs, the Ag NWs after UV/O_3_ treatment for various times, the graphene oxide (GO)-treated Ag NWs, the GO-treated Ag NWs after UV/O_3_ treatment for 30 min, and the GO film. GO flakes coated on the Ag NW electrode were introduced to reduce the degradation of the Ag NW electrode resulting from the UV/O_3_ treatment. In this structure, GO flakes with a size of about 5 μm covered a portion of the Ag NW electrode, resulting in a delay in the deterioration of the Ag NW caused by the UV/O_3_ treatment. The surface resistance of the Ag NW electrode without a GO treatment slightly decreased for UV/O_3_ treatment times less than 10 min, gradually increased with increasing treatment time, and then significantly increased for treatment times longer than 50 min, as shown in Fig. 8(a). While the sheet resistance of the GO-treated Ag NW electrode tended to increase slightly with increasing treatment time, its rate of increase was relatively small. We were not able to measure the sheet resistances in the two samples after UV/O_3_ treatment for 3 hr because the Ag NW network had been broken. However, these results show that GO treatment for the Ag NW electrodes can significantly reduce the deterioration of the Ag NW electrodes caused by using the UV/O_3_ treatment. The reduction in the degradation phenomenon was confirmed by using the absorption spectra shown in Fig. 8(b). The absorption spectra of the GO-treated Ag NW electrode were added to the absorption spectra of the conventional Ag NW electrode. The TSPR peak at 380 nm for the pristine Ag NW disappeared due to the UV/O_3_ treatment, but the TSPR peak from the GO-treated Ag NW did not disappear after the UV/O_3_ treatment. The reason the TSPR peak was observed after the UV/O_3_ treatment was that the TSPR generated in the Ag NWs continued to appear because the shape of the Ag NWs covered with the GO flakes had not changed. These results show that the simple process of GO treatment can sufficiently reduce the degradation phenomena due to UV irradiation. Even though the transmittance of the Ag NW electrode decreased from 94 to 88% resulting from the GO treatment, the simple GO treatment reduced the sheet resistance by 50% and improved the UV resistance of the Ag NW electrode. However, the structure that combines the Ag NW electrode with a protective film with a low UV transmittance needs to be investigated further in order to control the UV deterioration phenomenon, which, in turn, should prevent contact between nitrogen or oxygen atoms and the Ag NWs.

## Discussion

The degradation mechanisms of Ag NW electrodes caused by irradiating them with UV light were clarified based on variations in the electrical, optical, structural, and chemical properties. While the thermal treatment of the Ag NW electrodes produced only morphological variations in the Ag NWs, their treatment with UV rays was accompanied by a morphological change due to compositional changes on the surfaces of the Ag NWs. Even though the sheet resistances of the Ag NW electrodes at an initial stage decreased slightly with increasing UV/O_3_ treatment time due to the destruction of the PVP protective film, the Ag NW network was broken due to the combination and the decomposition of Ag and N or O atoms, resulting in a significant increase in the sheet resistance. The variation in the TSPR peak intensity due to the morphological change in the Ag NWs was confirmed by using the absorption spectra and the SEM images. XPS spectra demonstrated that thermal treatment produced only a shape variation in the Ag NWs without any chemical composition variation and that the degradation due to UV/O_3_ treatment was caused by a phase shift of Ag atoms and a sharp decrease in the numbers of C and N atoms, resulting in a morphological deformation of the Ag NWs due to the formation and the decomposition of silver nitride and silver oxide on the surfaces of the Ag NWs. The increase in the sheet resistance and the disappearance of the TSPR peak due to the structural degradation resulting from the UV/O_3_ treatment were reduced in the Ag NW electrodes coated with GO flakes. These results provide researchers with essential information for improving the stabilities of various flexible appliances utilizing Ag NW electrodes.
